# Image free-viewing as intrinsically-motivated exploration: estimating the learnability of center-of-gaze image samples in infants and adults

**DOI:** 10.3389/fpsyg.2013.00802

**Published:** 2013-10-31

**Authors:** Matthew Schlesinger, Dima Amso

**Affiliations:** ^1^Department of Psychology, Southern Illinois UniversityCarbondale, IL, USA; ^2^Cognitive, Linguistic, and Psychological Sciences, Brown UniversityProvidence, RI, USA

**Keywords:** visual exploration, perceptual development, intrinsic motivation, eye movements, image free-viewing

## Abstract

We propose that free viewing of natural images in human infants can be understood and analyzed as the product of intrinsically-motivated visual exploration. We examined this idea by first generating five sets of center-of-gaze (COG) image samples, which were derived by presenting a series of natural images to groups of both real observers (i.e., 9-month-olds and adults) and artificial observers (i.e., an image-saliency model, an image-entropy model, and a random-gaze model). In order to assess the sequential learnability of the COG samples, we paired each group of samples with a simple recurrent network, which was trained to reproduce the corresponding sequence of COG samples. We then asked whether an intrinsically-motivated artificial agent would learn to identify the most successful network. In Simulation 1, the agent was rewarded for selecting the observer group and network with the lowest prediction errors, while in Simulation 2 the agent was rewarded for selecting the observer group and network with the largest rate of improvement. Our prediction was that if visual exploration in infants is intrinsically-motivated—and more specifically, the goal of exploration is to learn to produce sequentially-predictable gaze patterns—then the agent would show a preference for the COG samples produced by the infants over the other four observer groups. The results from both simulations supported our prediction. We conclude by highlighting the implications of our approach for understanding visual development in infants, and discussing how the model can be elaborated and improved.

## Introduction

Within minutes of birth, human infants open their eyes and begin to explore the visual world (Slater, 2002). Although neonates lack visuomotor experience—and their visual acuity is poor—their eye movements are not random (Fantz, 1956; Haith, 1980). Instead, infants' gaze patterns are organized in a manner that facilitates the discovery and learning of relevant visual features and objects, such as the caretaker's face (e.g., Maurer and Barrera, 1981; Bushnell et al., 1989; Morton and Johnson, 1991).

With additional experience, infants not only gain further control over their eye movements, but their gaze patterns also continue to develop. For example, during the first month after birth, infants tend to limit their scanning to a small portion of an image (Bronson, 1982, 1991). By age 3 months, however, infants produce gaze patterns that are more systematically distributed over visual scenes. During the same age period, comparable changes also occur in a number of other related visual skills, such as maintaining fixation of a target object in the presence of distracting stimuli, as well as selecting informative regions of the visual scene to fixate and encode (e.g., Johnson et al., 2004; Amso and Johnson, 2005).

There have been several important advances in the study of infants' gaze patterns. One approach leverages the tendency for infants to orient toward salient, predictable events, and in particular, events that are contingent on infants' own actions (e.g., Haith et al., 1988; Kenward, 2010). For example, Wang et al. (2012) recently developed a gaze-contingent paradigm in which infants quickly learned to anticipate the appearance of a picture that was “triggered” by first fixating an object at another location. This work highlights the fact that infants' visual-activity is prospective and future-oriented.

A second advance is the use of image free-viewing methods, which record and analyze infants' eye movements as they view a series of images or video clips, often including naturalistic scenes (e.g., Aslin, 2009; Frank et al., 2009, 2012). In contrast to methods that present an implicit task to the infant, such as comparing two images or locating a target object, image free-viewing is comparatively less-constrained, and may more accurately reflect not only infants' spontaneous gaze patterns, but also the process of information pickup and learning that occurs in real time during visual exploration. While early work using image-free viewing tended to rely on somewhat coarse analytical methods, such as comparing time spent viewing specific regions of interest (ROIs; e.g., Bronson, 1982, 1991), more recent work in this area has employed relatively sophisticated quantitative methods. For example, Frank et al. (2009) computed the frame-by-frame image saliency of a short animation clip (i.e., “A Charlie Brown Christmas”), and then compared infants' attention to faces in the clip vs. their attention to high-salience non-face regions. A key finding from their analysis was that at age 3-months, infants' gaze patterns were more strongly influenced by salience than by social stimuli such as faces; however, by age 9 months, this pattern reversed, and infants oriented reliably to faces.

Finally, the approach we propose here represents a third advance. In particular, there are several recent models that successfully capture the kinematic properties of infants' gaze patterns during conventional tasks, such as preferential looking, gaze following, and visual search (e.g., Schlesinger et al., 2007; Triesch et al., 2007; Perone and Spencer, 2013). However, to our knowledge, our model is the first attempt to apply incremental, adaptive-learning methods (i.e., artificial neural networks and reinforcement learning) as a computational tool for analyzing infants' gaze patterns during image free-viewing.

Specifically, we propose that in addition to analyzing the spatial distribution and timing of infants' gaze patterns, the *sequential content of their fixations during image free-viewing* may also provide an important source of information. In particular, the sequence of fixations produced by an observer can be interpreted as a series of high-resolution visual samples, each centered at the corresponding gaze point (i.e., center-of-gaze or COG samples; Dragoi and Sur, 2006; Mohammed et al., 2012). As a form of exploration in the visual modality, these COG samples are similar to the tactile data generated by structured hand and finger movements during haptic object exploration (i.e., exploratory procedures or EPs; Klatzky and Lederman, 1990), insofar as different sampling patterns are the result of different exploration strategies.

In this paper, we propose that infants' gaze patterns during image free-viewing are a form of visual exploration, and that the sequential structure embedded within these patterns can be analyzed with the theoretical framework of *intrinsic motivation*. More specifically, we suggest that:

**Learning objective 1**: over the short term (i.e., real time), the goal of visual exploration is to accurately predict the content of the next fixation (i.e., the subsequent COG sample), given the current fixation together with the history of recent fixations.
**Learning objective 2**: superimposed on the timescale of learning objective 1, a longer-term goal of visual exploration is to learn how to generate sequentially learnable gaze patterns, that is, to learn how to scan images or scenes such that the resulting set of COG samples is sequentially predictable.

Learning objective 1 is predicated on the idea that prediction-learning and future-oriented actions are pervasive characteristics of infant development (e.g., Haith, 1994; Johnson et al., 2003; von Hofsten, 2010). In addition, a related mechanism that may underlie prediction-learning is the detection of statistical patterns or regularities in the environment, such as those in linguistic input or natural scenes (e.g., Field, 1994; Saffran et al., 1996). However, a unique aspect of our proposal is that, rather than passively observing sensory patterns in the external world, infants may also contribute to the process of pattern detection by embedding structure in their own exploratory behavior.

The rationale for learning objective 2, meanwhile, is that in addition to acquiring specific skills, such as learning to grasp or walk, infants also engage in behaviors that seem to have no explicit purpose, such as babbling or playing with blocks. In other words, *intrinsically-motivated* behaviors are done simply for the sake of learning (Oudeyer and Kaplan, 2007; Baldassarre and Mirolli, 2013; Schlesinger, 2013). This contrasts with *extrinsically-motivated* behaviors, which have a clear and (typically) biological benefit, such as obtaining food, rest, or sex (Baldassarre, 2011).

By this view, we argue that visual exploration serves two developmental functions. First, at the moment-to-moment level (learning objective 1), infants learn to discover and predict the particular statistical regularities of the images and scenes they are scanning (e.g., moving objects tend to remain on continuous trajectories, natural scenes are typically illuminated from above, “angry” eyes tend to co-occur with a frowning mouth, etc.). Second, and over a longer timescale (learning objective 2), infants are also “learning to learn,” that is, their scanning strategies are refined, and in particular, infants are improving in their ability to detect and attend to relevant visual features. In our model, we conceptualize this second-order learning process as an intrinsically-motivated artificial agent, which observes the performance of five scanning strategies, and is rewarded for selecting the strategy that produces the lowest (or most rapidly falling) prediction errors.

In order to pursue the first learning objective, we assigned five unique sets of COG samples to each of five simple recurrent networks (SRNs). We selected the SRN architecture as a computational tool for two specific reasons. First, it serves as a proxy for the statistical-learning mechanism noted above. In particular, it is well-suited to detecting regularities or statistical dependencies within temporal sequences of input. Second, we also exploited SRNs as a means to measure the relative predictability of the sequences produced by the observer groups. Specifically, the training errors produced by the SRN provide a straightforward metric for assessing learnability of the COG samples.

Each set of COG samples was generated by a different group of real or artificial observers: 9-month-olds, adults, an image-saliency model, an image-entropy model, and a random-gaze model. The task of each SRN is to learn to reproduce the sequence of COG samples produced by its corresponding group. We then pursued the second learning objective by creating an intrinsically-motivated artificial agent, which selects among the five SRNs as they are trained, and is rewarded for either selecting the SRN with the lowest errors (Simulation 1), or the SRN that learns the fastest (Simulation 2). We return to this issue below, where we describe the specific reward functions used to evaluate the choices of the intrinsically-motivated agent.

We reasoned that each group of real or artificial observers collectively represents a distinct scanning pattern or strategy, and as a result, the COG samples generated by each group should be differentially learnable. In addition, given our proposal that infants' visual exploration is specifically geared toward the goals of (1) sequential predictability and (2) optimal prediction-learning, we therefore, hypothesized that the COG samples produced by 9-month-olds would be selected first by an intrinsically-motivated agent, whether the reward function is based on learning errors (Simulation 1) or change in the rate of learning (Simulation 2). We also predicted that as reward diminishes in Simulation 2 (i.e., as learning of the infants' COG samples asymptotes), the agent should then shift its preference from the infants' COG samples to the adults' samples. This was an exploratory prediction, based on the assumption that adults' gaze patterns are not only influenced by sequential learnability (like infants), but that they are also informed by the observer's history of goal-directed activity (e.g., Shinoda et al., 2001; Hayhoe and Ballard, 2005).

The rest of the paper is organized as follows. We first describe the set of images presented to the five groups of observers, as well as the procedure used to acquire the gaze data from the human observers. We also describe the design of the three groups of artificial observers, and the analogous procedure used to generate the gaze data from each of these groups. We conclude this section by explaining how the gaze data were used to generate COG samples. In the next section, we then describe the architecture and learning algorithms used in the SRN prediction networks (PNs) and the intrinsically-motivated agent. Following this, we present Simulation 1, in which the artificial agent vicariously explores the COG samples by selecting among the five SRNs, and learns by trial-and-error to find the SRN with the lowest prediction errors. Next, in Simulation 2 we present the findings of a closely-related reward function, in which the agent is rewarded for finding the SRN with the fastest learning progress (i.e., the largest decline in the error rate over successive training epochs). In the final section, we relate our findings to the development of visual exploration in infants, and describe some ways to address the limitations of our current modeling approach.

## Materials

### Test images

**Figure 1:**
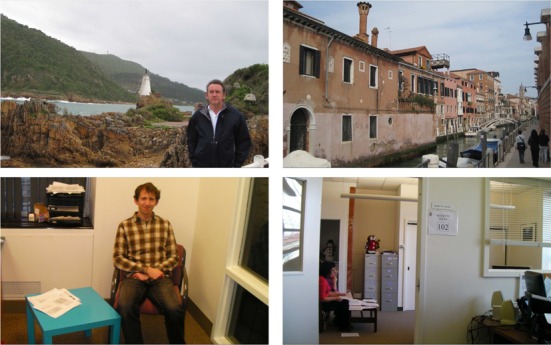
**Four of the test images**.

Sixteen naturalistic, color images were used as stimuli for collecting eye movements, including 8 indoor and 8 outdoor scenes. One or more people were present in each image; in some images, the people were in the foreground, while in others they were in the background. Figure 1 presents 4 of the 16 test images. The infant and adult observers were presented with the test images at the original image resolution (1680 × 1050 pixels), while the artificial observers were presented with downscaled versions of the images (480 × 300 pixels). As we note below, all of the infant and adult fixations were rescaled to the lower resolution, so that real and artificial observers' gaze data could be directly compared.

### Observer groups

#### Real observers

Eye-movement data were collected from 10 adults and 10 9-month-olds infants (mean ages = 19 years and 9.5 months, respectively). Except where noted, a comparable procedure was used for testing both adult and infant participants. All participants provided either signed consent for the study, or in the case of the infants, assent was provided by the infants' parents.

Participants sat about 70 cm from a 22” (55.9 cm) monitor. Infants sat in a parent's lap. Eye movements were recorded using a remote eye tracker (SMI SensoMotoric Instruments RED system). In addition, a standard digital video camera (Canon ZR960) was placed above the computer screen to record children's head movements. All calibration and task stimuli were presented using the Experiment Center software from SMI. Before beginning the task, point-of-gaze (POG) was calibrated by presenting an attractive, looming stimulus in the upper left and lower right corners of the screen. The same calibration stimulus was then presented in the four corners of the screen in order to validate the accuracy of the calibration.

We eye tracked participants as they freely scanned 16 color photographs depicting both indoor and outdoor scenes (see Figure 1 for examples; for a comparable procedure, see also Amso et al., 2013). All images were presented for 5 s and spanned the entire display. The order of image presentation was randomized. A central fixation target was used to return participants' POG to the center of the screen between images.

#### Artificial observers

The purpose of creating the artificial observers was to generate a set of synthetic gaze patterns, in which the underlying mechanism driving gaze from one location to the next was known in advance. In addition, the three groups of artificial observers also provide a well-defined baseline for comparison with the infant and adult observers (see Frank et al., 2009, for a similar approach).

***Saliency model***. The saliency model was designed to simulate an artificial observer whose gaze pattern is determined by bottom-up visual features, such as edges or regions with strong light/dark contrast. In particular, each test image was transformed by first creating three feature maps (tuned to oriented edges, luminance, and color contrast, respectively), and then summing the feature maps into a saliency map. We then used each saliency map to generate a series of simulated fixations.

**Figure 2:**
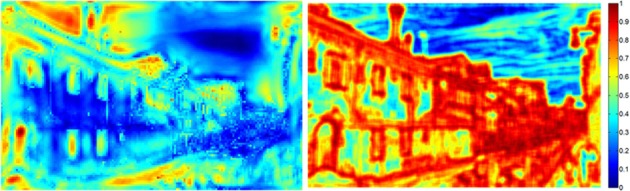
**Examples of corresponding saliency and entropy maps (left and right images, respectively) used to simulate gaze patterns in the artificial observer groups (compare to original image in Figure 1)**. The color legend on the right illustrates the range of possible values for each map.

1. **Feature maps**. The original images were first downscaled to 480 × 300. Next, each image was passed through a bank of image filters, resulting in three sets of feature maps: 4 oriented edge maps (i.e., tuned to 0°, 45°, 90°, and 135°), 1 luminance map, and 2 color-contrast maps (i.e., red-green and blue-yellow color-opponency maps). In addition, this process was performed over 3 spatial scales (i.e., to capture the presence of the corresponding features at high, medium, and low spatial frequencies), by successively blurring the original image and then repeating the filtering process [for detailed descriptions of the algorithms used for each filter type, refer to Itti et al. (1998) and Itti and Koch (2000)]. As a result, 21 total feature maps were computed for each test image.
2. **Saliency maps**. The saliency map was produced by first normalizing the 21 corresponding feature maps, and then summing them together. For the next step (simulating gaze data), each saliency map was downscaled to 48× 30. These resulting maps were then normalized, by dividing each map by the average of the highest 100 saliency values from that map. Figure 2 illustrates the saliency map (left image) for one of the outdoor scenes (compare with the original image in Figure 1).
3. **Simulated gaze data**. In order to equate the mean number and frequency of gaze shifts across the real and artificial observers, the gaze data of the infants and adults were pooled, and the corresponding values were computed. This resulted in a mean of 13 fixations per image, and a mean latency of 300 ms between fixations. For the artificial observers, the simulated timestep was 33 ms per processing cycle (i.e., 30 updates per second). These values were then used as fixed parameters for the artificial observers. A single trial was simulated by iteratively updating a fixation map—which is the difference between the saliency map and a decaying inhibition map (see below)—and selecting a location on the fixation map every 300 ms. Note that the inhibition map served as an analog for an inhibition-of-return (IOR) mechanism, which allowed the saliency model to release its gaze from the current location and shift it to other locations on the fixation map.

Each trial began by selecting the initial fixation point at random. Next, the inhibition map was initialized to 0, and a 2D Gaussian surface was added to the map, centered at the current fixation point, with an activation peak equal to the value at the corresponding location on the saliency map. Over the subsequent 300 ms, activity on the inhibition map decayed at a rate of 10% per timestep. At 300 ms, the next fixation point was selected: (a) the fixation map was updated by subtracting the inhibition map from the saliency map (negative values were set to zero), (b) the top 100 values on the saliency map were identified, and (c) the saliency value at each of these locations was converted to a probability using the softmax function:

(1) $$\text{Probability of selection}=e^{s/\tau }/\sum_{i=1}^{100}e^{s_{i}/\tau }$$

where *s* is the given saliency value, and τ is the temperature parameter (fixed at 1). One of these 100 locations on the fixation map was then chosen stochastically, as a function of the corresponding probability values.

This process of updating the inhibition and fixation maps and selecting a new fixation point continued until 13 fixations were performed. The gaze data for 10 artificial observers from the saliency group were then simulated by sweeping through the set of 16 images, once per each observer, and then repeating the process 10 times. It is important to note that repetitions of the simulation process over the same image resulted in distinct gaze patterns, due not only to randomization of the initial fixation, but also to stochasticity in the procedure for selecting subsequent fixations.

***Entropy model***. The entropy model simulated an artificial observer whose gaze pattern is determined by image “information,” that is, by the presence of structured or organized visual patterns within the image (e.g., Raj et al., 2005; Lin et al., 2010). As a proxy for information, image entropy was estimated for each image. In particular, image entropy reflects the computational cost of compressing an image, based on the frequency of repeated pixel values. The function used for computing image entropy was:

(2) $$\text{Image entropy}=-\sum_{i=1}^{256}p_{i}*\log_{2}(p_{i})$$

where the original image is converted to grayscale, pixel values are sorted over 256 bins, and *p* represents the proportion of pixels in each bin.

1. **Entropy maps**. Comparable to the saliency maps, the entropy maps were produced by first downscaling the original images to 480 × 300 and then converting them to grayscale. Note that the image entropy function produces a single scalar value over the entire image. Thus, the entropy map was produced by sweeping an 11 × 11-pixel window over the grayscale image, and replacing the pixel value at the center of the window with the corresponding entropy value for that 11 × 11 square. Figure 2 illustrates the entropy map (right image) for one of the outdoor scenes (compare with the original image in Figure 1).
2. **Simulated gaze data**. Once the entropy maps were computed for the set of 16 test images, they were then downscaled a second time and normalized, using the same process as described above for the saliency maps. Finally, gaze data for 10 simulated observers were generated, also using the same procedure as described above.

***Random model***. The random model was designed as a control condition, to simulate the gaze pattern of an observer who explored the test images by following a policy in which all locations are equally-likely to be selected. Thus, no maps were produced for this group. Instead, 2080 x- and y-locations were chosen at random (i.e., 13 fixations × 16 images × 10 observers).

**Figure 3:**
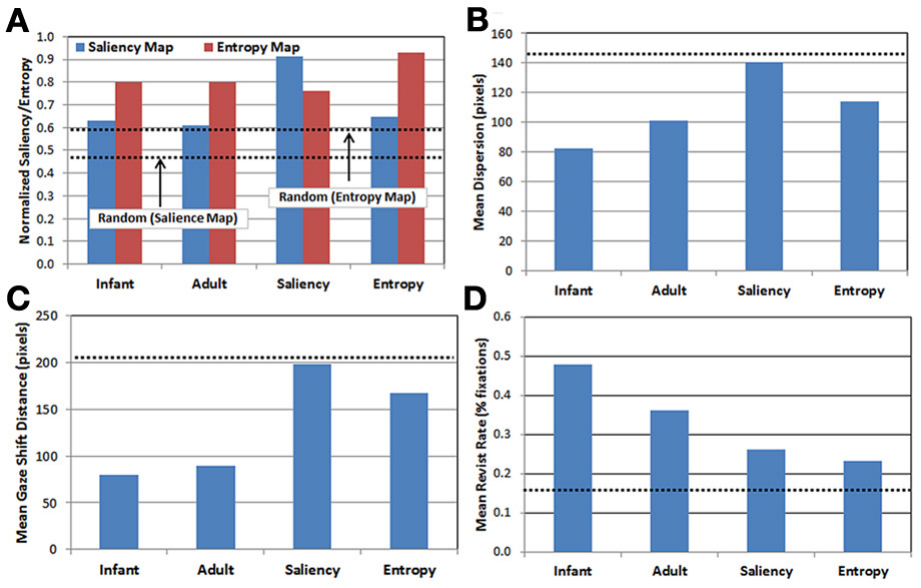
**Comparison of gaze patterns across the 5 observer groups (see text for details)**. **(A)** Mean map values calculated by projecting each group's gaze points on to the saliency (blue) and entropy (red) maps, respectively; **(B)** mean dispersion (spread) of fixations; **(C)** mean gaze shift distance; and **(D)** mean proportion of revisits. Dashed lines represent performance of the random observer group.

***Descriptive statistics***. We briefly compare here the gaze data produced by each of the five observer groups. In all cases, note that because the random group provides a baseline estimate of performance at chance level, the results from this group are plotted in Figure 3 as dotted lines (rather than as bars). Figure 3A presents the results of projecting each observer group's fixations onto the saliency and entropy maps, respectively, and then computing the average saliency (blue bars) and entropy values (red bars) for the corresponding fixation locations. This analysis provides a measure of the relative influence of saliency vs. entropy for each group's scan patterns. In particular, higher mean values reflect a tendency to orient toward regions in the image with higher levels of saliency and/or entropy, respectively (recall that the values on each map were normalized between 0 and 1). Note that the upper dashed line in Figure 3A represents the mean normalized entropy produced by the random observer group, while the lower dashed line represents mean normalized saliency for the same group.

There are three important results. First, as expected, the saliency and entropy observer groups produce near-maximal values (i.e., 90%) for their respective maps. Second, for both infants and adults, the gaze patterns resulted in higher mean levels of entropy than salience. Third, even for the random group, the same pattern was also true. As Figure 2 suggests, this may be due to differences in how saliency and entropy are distributed over each image—that is, saliency was sparsely distributed while entropy was relatively broadly distributed.

In addition, Figures 3B–D present the results of three kinematic measures. First, Figure 3B plots the mean dispersion of fixations for each group. Dispersion was computed by first calculating the centroid of the fixations (i.e., the mean fixation location) within each trial, and then calculating the mean distance of the fixations within that trial from the centroid. As Figure 3B indicates, infants tended to have the least-disperse gaze patterns, followed by adults. Interestingly, the dispersion of fixations produced in the saliency observer group was nearly the same as the random observer group.

Next, Figure 3C presents the mean gaze shift distance for each group. This distance was calculated by computing how far the fixation point traveled (in pixels) from each fixation to the next. Like the previous result, infants produced the shortest gaze shift distance, again followed by adults. Similarly, the saliency observer group produced gaze shift distances similar to the random observer group, while the entropy observer group had gaze shift distances that fell midway between the real and artificial observers.

Finally, Figure 3D presents the mean revisit rate for each observer group. Revisit rate was estimated by first creating a null frequency map (a 480 × 300 matrix with all locations initialized to zero). Next, for each fixation, the values within a 41 × 41 square (centered at the fixation location) on the frequency map were incremented by 1. This process was repeated for all of the fixations within a trial, and the frequency map was then divided by the number of fixations. For each trial, the maximum value from this map was recorded, reflecting the location in the image that was *most frequently* visited (as estimated by the 41 × 41 fixation window). The maximum value was then averaged across trials and observers within each group, providing a metric for the peak proportion of fixations that a particular location in each image was visited, on average. As Figure 3D illustrates, a key finding from this analysis is that infants have the highest revisit rate (nearly 50%), while all three of the artificial observer groups have the lowest rates.

### COG image samples

To maintain tractability of the training set for the SRNs, we randomly selected 20 trials from each group of observers. Selection was subject to several constraints, including: (1) within a group, each observer contributed 2 trials (i.e., gaze data for 2 images), and (2) selection of the corresponding images was counterbalanced both within observer groups and across the 16 images (each image was selected as equally-often as possible across groups). Once the specific trials/images were selected for each group, the gaze data (i.e., sequences of fixation points) were then used to generate the COG training stimuli.

Specifically, for a given observer and trial, a 41 × 41 grayscale image—centered at the first fixation point—was sampled from the corresponding test image. The dimensions of the COG sample were derived from the display size and viewing distance of the live observers, and correspond to a visual angle of 1.6°, which falls within the estimated range of the angle subtended by the human fovea (Goldstein, 2010). This sampling process continued for the second fixation point, and so on, until the number of fixations for that observer and trial was reached. The process for obtaining the COG samples for a single trial was then repeated through each of the five observer groups, resulting in 20 trials of COG samples per group (with an average of 13 samples per trial, or approximately 260 samples per group).

**Figure 4:**
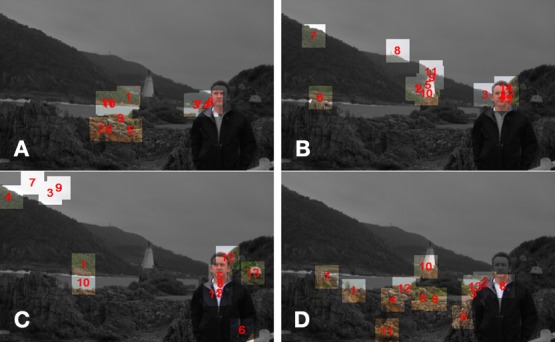
**Illustration of the COG samples produced during a single trial with test image 4, in the infant (A), adult **(B)**, saliency **(C)**, and entropy **(D)** observer groups (non-fixated areas are darkened)**.

To help illustrate how a typical set of COG samples appears in relation to its corresponding test image, Figure 4 presents the samples produced during a single trial (with test image 4), in the infant, adult, saliency, and entropy observer groups, superimposed on to the respective test image. Consistent with Figure 3B, note that the infant's fixations tend to fall into two spatial clusters, while the adult's fixations are more disperse.

## Model architecture and learning algorithms

**Figure 5:**
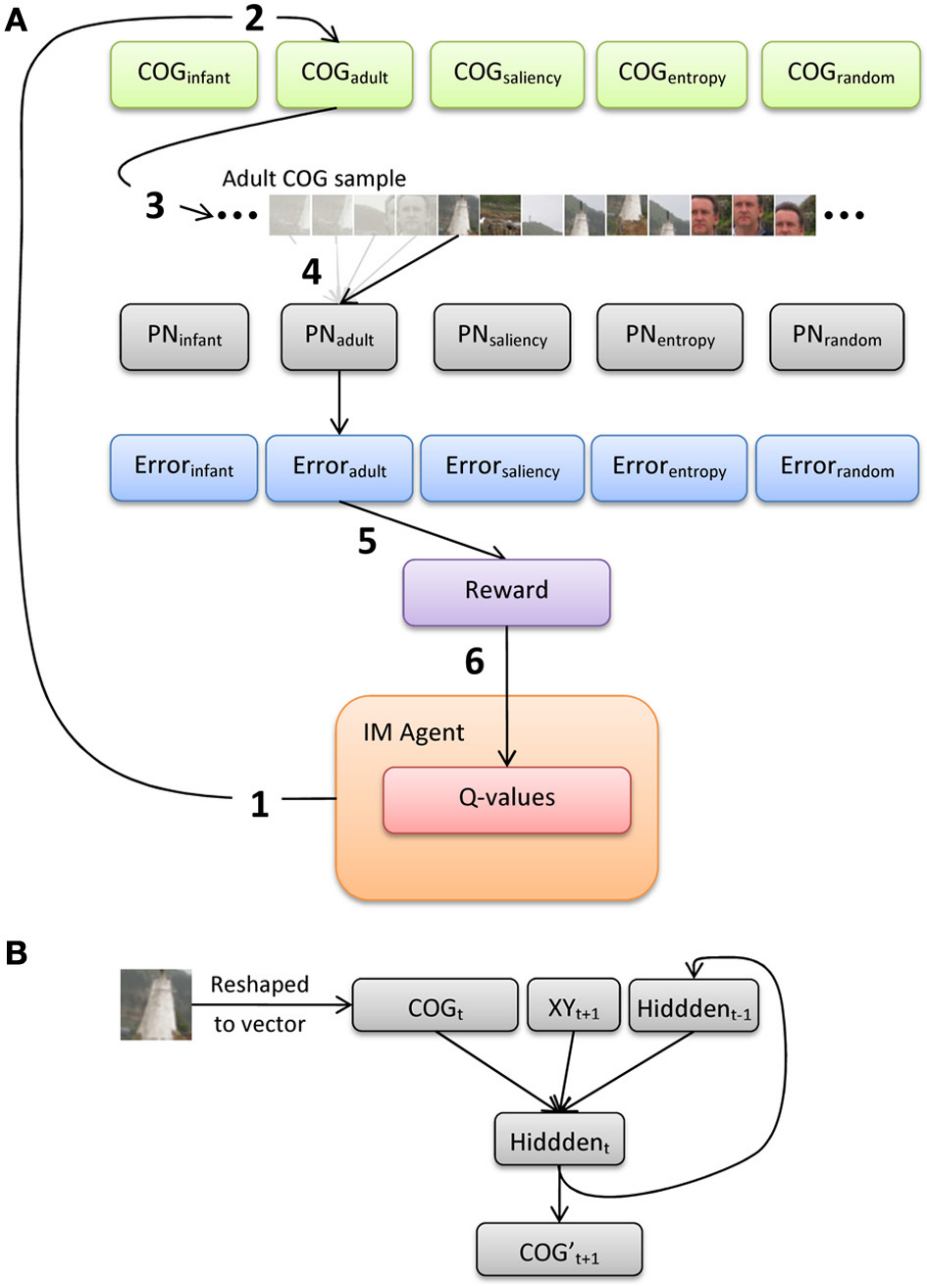
**(A)** Illustration of the processing pathway through the model during a single episode, and **(B)** architecture of the prediction networks (PNs).

Figure 5 illustrates an overview of the model architecture, which implements a conventional reinforcement-learning model layered over a bank of recurrent neural networks. We first provide here a general description of the six major processing steps in the model, and present below a more-detailed description of the PNs and the intrinsically-motivated artificial agent (IM agent).

The IM agent learns over a series of discrete episodes. At the start of each episode (Figure 5A, step 1), the IM agent first selects one of the five observer groups. This choice is intended to represent an analog for presenting an image to an observer, who then explores the image by choosing from a set of distinct gaze or scanning “strategies” (alternatively, these strategies could be described as learning goals, behavior or action patterns, etc.). In particular, the IM agent has no direct knowledge of how each strategy is designed or how it operates. Rather, the IM agent bases its decision simply on the current set of *Q*-values for the set of five choices, which each estimate the long-term sum of rewards expected to result from selecting the corresponding choice. Once one of the gaze-pattern strategies (i.e., observer groups) is selected, the COG samples from the corresponding group of observers are retrieved. For example, in Figure 5A, the IM agent selects the adult observer group (step 2).

At the next processing step, the 20 sets of COG samples (from the selected observer group) are then presented to the corresponding SRN (step 3; note that only 1 of the 20 sets is illustrated here). In particular, we implement a bank of five SRNs, each of which is devoted to a single observer group, in order (a) to maintain learnability estimates of all five groups in parallel, and (b) to avoid the risk of catastrophic interference by training a single network on the COG samples from all five groups. We refer to the SRNs as PNs, as they are explicitly trained to reproduce the series of 41 × 41 samples, one at a time. In the case of Figure 5, one of the 20 COG sample sets is selected at random from the adult observer group, and the first sample from this set is presented to PN_adult_. The output of the network is its “prediction” of the second sample (properly speaking, since training is offline, i.e., after the samples were collected, the PN learns to *reproduce* a sequence that is iteratively presented). After each output, a training signal is computed using backpropagation-of-error and used to adjust the PN's connection weights. This continues until all of the COG samples in the observer group have been presented to the PN (step 4).

At step 5, the average prediction error for the previous training sweep is computed, and then transformed into a scalar reward value. As we highlight below, we investigate two reward functions: reward based on the magnitude of error (i.e., reward is inversely related to error), and reward based on learning progress (i.e., reduction in error over two consecutive sweeps through the COG samples in an observer group). During the final processing step (6), the new reward value is used to update the set of *Q*-values, and the IM agent makes its next selection.

### Prediction networks

Each PN is a standard 3-layer Elman network, with recurrent connections from the hidden layer back to the input layer (i.e., context units; Elman, 1990). In particular, the PN implements a forward model, in which the current sensory input (plus a planned action) is used to generate a prediction of the next expected input (e.g., Jordan and Rumelhart, 1992). Prior to training the PN, each of the COG samples is converted to grayscale values between 0 and 1. As Figure 5B illustrates, the input layer is composed of 2083 units, including a vector of 1681 units that encode the grayscale pixel values of the COG sample, 2 units that encode the (normalized) x- and y-coordinates of the upcoming COG sample, and 400 context units (which copy back the activity of the hidden layer from the previous time step). There are 400 units in the hidden layer (i.e., roughly 75% compression of the input) and 1681 output units.

All connections in the PN are initialized with random values between 0 and 1, which are then divided by the number of incoming units (i.e., fan-in). For each simulation run, the same PN is cloned five times, so that all five PNs begin with the same set of initial connection weights. As noted above, each PN is presented with only the COG samples from its corresponding observer group. Once an observer group is selected by the IM agent, the 20 COG sample sets are then presented to the appropriate PN in random order. Recall that each set of COG samples represents the gaze data from a single observer and a single trial. In order to remove the influence of previous trials on the context layer activation, the units in the context layer of the PN are initialized to 0.5 at the start of each trial. A single training epoch is defined as a sweep through all 20 trials.

Prediction error is measured as the root mean-squared error (RMSE), computed over the difference between each predicted and observed next COG sample, and then averaged over the entire trial. Mean trial errors are then averaged together over the 20 trials; this value represents the mean prediction error for the IM agent's current episode, and is used to compute the reward signal.

### IM agent

The IM agent simulates a naïve, active observer that is reinforced for visually exploring its environment. As Figure 5 illustrates, the IM agent is provided with the opportunity to select among five predefined sets of visual samples and a corresponding PN, each of which represents (ostensibly) a unique scanning experience and learning episode over the set of 16 test images. After each selection, the IM agent receives a reward signal as feedback that is proportional—not to the content or the quality of the chosen gaze samples *per se*—but rather, to the relative success of the chosen PN in predicting the resulting sequence of COG samples. In other words, the IM agent is rewarded for choosing the set of COG samples (i.e., a pattern of visual exploration) that is learned optimally.

In principle, defining an *exploration reward* on the basis of *learnability* runs the risk of generating an unintended outcome. For example, one way to maximize the performance of the PN is to hold the fixation point constant, that is, to continue looking at the same location. Such a strategy, however, also provides limited visual information (i.e., it maximizes prediction but minimizes exploration). At the other extreme, a completely random gaze sequence may be highly informative, but difficult, if not impossible to predict. Given the putative goal of visual exploration, therefore, a reasonable trade-off is to select a gaze sequence that is both informative *and* predictable (i.e., varied but also systematically structured). We therefore, note here that linking the reward function to prediction learning captures an important dimension of visual exploration, but that other facets such as novelty are also likely to play a role (for a comprehensive discussion of knowledge-based vs. competence-based approaches to intrinsic motivation, see Oudeyer and Kaplan, 2007, and Baldassarre and Mirolli, 2013).

Because the actions selected by the IM agent are influenced by the performance of the PNs, there are effectively two timescales: an “inner loop,” which is defined as presenting the selected PN with the COG samples from a single trial, and the “outer loop,” which is a single episode and is defined as the IM agent's selection of an observer group, a training epoch of the corresponding PN, the generation of an intrinsic reward signal, and the updating of the IM agent's *Q*-values (as illustrated in Figure 5). For both Simulations 1 and 2, therefore, a single simulation run included 500 iterations of the outer loop (i.e., episodes). In addition, recall that during each iteration of the outer loop, there were 20 iterations of the inner loop for the selected PN.

As we highlight below, the objective or reward function that we implemented was varied across simulations. In Simulation 1, the reward was defined as:

(3) $$r_{t}=1-{\text{Error}}_{t}$$

where *r*_*t*_ is the reward received for the *t*th iteration of the outer loop, and Error_*t*_ is the mean error produced by the PN selected during iteration *t*. This function therefore, rewards the IM agent for selecting the observer group with the lowest prediction errors (compare to “predictive novelty,” i.e., Equation 9 in Oudeyer and Kaplan, 2007). In contrast, during Simulation 2 the reward function was defined as the percent change in prediction error over two consecutive iterations of the inner loop:

$$r_{t}=({\text{Error}}_{t-1}-{\text{Error}}_{t})/{\text{Error}}_{t-1}$$

where Error_*t*_ is defined as in Equation (3), and Error_*t* − 1_ represents the corresponding mean error from the previous iteration. Note that in this case, each time a PN was selected, it was trained for two consecutive epochs before the IM agent received a reward.

Two steps were implemented to ensure that the IM agent sufficiently explored each of the five observer groups. First, at the start of each simulation run, the IM agent's *Q*-values were initialized optimistically, that is, they were set to initial values higher than were expected to occur during learning. Second, the Softmax function [see Equation (1)] was used for action selection, which provided an additional source of stochasticity and variability into the IM agent's choice of observer group.

After selecting an observer group and receiving a reward for the selection, the IM Agent's *Q*-value for that group was updated. The update rule implemented was:

(4) $$Q_{t}=Q_{t\;-1}+\alpha (r_{t}-Q_{t-1})$$

where *Q*_*t* − 1_ is the *Q*-value for the selected observer group before the most recent iteration of the inner loop, and *Q*_*t*_ is the new, updated value after the iteration. Finally, α represents the learning rate, which was fixed for each simulation.

## Simulation 1

In Simulation 1, the IM agent vicariously explored the 16 test images by repeatedly selecting from a set of COG samples, each of which captured the process of scanning the images in either real or simulated real time. After each selection, the IM agent then received a reward which represented the relative ease or difficulty of sequentially predicting the selected gaze samples. In particular, the IM agent received a larger reward when it picked a set of COG samples that were “easily” learned (i.e., that resulted in comparatively lower prediction errors), while the scalar reward was lower when the COG samples (and the corresponding PN) produced higher prediction errors. Our primary prediction was that, given the assumption that infants are mastering the skill of visual exploration, the COG samples produced by the 9-month-olds would be the most predictable, and therefore, the IM agent would prefer samples produced by the 9-month-olds over those from the other four observer groups.

### Method

Ten simulation runs were conducted. At the start of each run, the five PNs were initialized as described above. In addition, the set of *Q*-values for the five corresponding actions was uniformly initialized to 1. During Simulation 1, the temperature parameter τ used in the Softmax function for action selection was 0.01. Finally, the learning rate value α used for updating the *Q*-values (Equation 5) was 0.1. Each simulation run was composed of 500 episodes, during each of which the IM agent chose a set of COG samples, the corresponding PN was trained on the selected set of samples for one epoch, and the IM agent then received a reward and the respective *Q*-value was updated.

### Results

For the purpose of analysis, the results over the 10 simulation runs were averaged together. We focus here on three questions. First, during learning, does the IM agent develop a preference for any of the five observer groups? Second, how does the IM agent distribute its selections over the five groups? Finally, how well do the five PNs collectively perform over the 500 episodes?

**Figure 6:**
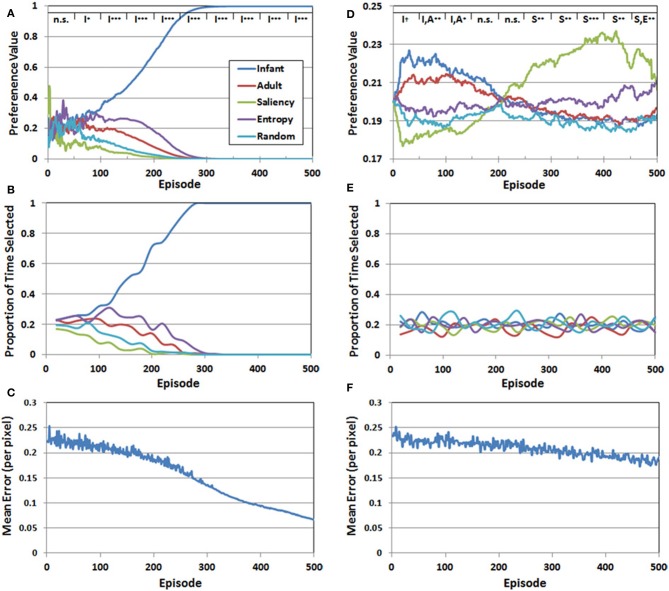
**Set of 3 performance measures for Simulation 1 (A–C) and Simulation 2 (D–F)**. The legend at the top of panels **(A,D)** represents the results of planned comparisons between the observer groups (n.s. = not significant, ^†^< 0.06, ^*^< 0.05, ^**^< 0.01, ^***^< 0.001). See the text for additional details.

We addressed the first question by transforming the *Q*-values at the end of each episode into standardized “preference” values, which are simply the probabilities assigned to the choices by the Softmax function. Figure 6A presents the mean preferences for the five observer groups as a function of episode, averaged across 10 simulation runs. Mean preferences were analyzed statistically by dividing the 500 training episodes into 10 blocks, each 50 episodes long. We then conducted a two-factor mixed-model ANOVA for each of the blocks, with observer group (infant, adult, saliency, entropy, and random) as the between-subjects factor, and episode as the within-subjects factor. We report here the results of the planned paired-comparison tests for the five observer groups, focusing specifically on whether the group (or groups) with the highest preference values differed significantly from the remaining observer groups. Note that the top legend in Figure 6A illustrates the outcome of these comparisons for each of the 50-episode blocks, by indicating the group/groups with the highest preference value and the significance level of the planned comparison (I = infant, A = adult, S = saliency, E = entropy, R = random).

There were three major findings. First, for approximately the first 50 episodes, preference values varied considerably, resulting in no significant differences between the five observer groups. Second, a preference for the COG samples from the infant observer group emerged between episodes 50 and 100, while the values for the other four groups continued to decline. Third, and confirming our prediction, this pattern continued and strengthened between episodes 100 and 500.

Figure 6B presents the proportion of time that each of the five observer groups was selected over the 500 episodes. Recall that because a stochastic decision rule was used to select the groups, the actual frequency of selection may not necessarily align with the corresponding preference values. However, as Figure 6B illustrates, there was a close match between the IM agent's preference values, and the resulting selection pattern. In particular, during the last 200 episodes, effectively all of the training time was directed toward the infant observer group's PN.

Finally, Figure 6C presents the RMSE—pooled over the five PNs—as a function of episode. At the start of training, the RMSE was approximately 0.25 per pixel. Fluctuations in the error level, between episodes 1 and 300, reflected the fact that the IM agent continued to explore the observer groups throughout this period. However, as the infant observer group became the sole preferred choice, the IM agent focused on the COG samples from this group and the error rate declined more consistently. By 500 episodes, the RMSE had fallen below 0.07. Thus, Figure 6C suggests that all of the PNs improved during training, but the infant group's PN eventually received the majority of training time and accordingly benefited.

## Simulation 2

While Simulation 1 confirmed our prediction that the IM agent would prefer the infant observer group's COG samples, it is also important to note that the particular reward function used potentially suffers from a “snowball” bias. In other words, because the reward function favored low prediction errors, the group with the lowest errors at the start of training would have an advantage over the other four groups. In addition, a bias toward providing this group with additional training time would then continue to improve the predictions of their PN, thereby lowering prediction errors further and increasing the advantage of that group. Such a bias would also reduce exploration of the competing groups, and consequently, leave them with higher errors.

To address this issue, we investigated an alternative reward function, which favored learning progress, that is, a reduction in the RMSE over two consecutive episodes. As Equation 4 highlights, the reward function in Simulation 2 was scaled by the RMSE of the first episode of each pair, which effectively produced a reward value equal to the percent change in the RMSE. Interestingly, this solves one problem while creating a new challenge for the model: in particular, by linking reward to *changes* in performance of the PNs, the IM agent's learning task becomes non-stationary. Specifically, by selecting the “best” (i.e., most-improving) observer group for training, learning in that group should eventually level off, and thus, the IM agent's long-term estimates of the group's *Q*-value should systematically drift downward over time. Fortunately, there is also a hidden advantage to this approach, namely, that the IM agent should therefore, switch its preference from the COG samples of one observer group to another, as improvement in the leading group slows. As we highlight in the discussion, such a switching pattern has the potential to be interpreted as a developmental pattern, in which the simulated observer shifts from one visual-exploration strategy to another.

Recall that our prediction for Simulation 2 was that, like Simulation 1, the COG samples from the infant observer group would be preferred first, and that the model would then shift its preference to the samples from the adult observer group.

### Method

The same procedures as Simulation 1 were followed in Simulation 2. However, given an expected decline in the absolute magnitude of the reward (relative to Simulation 1), the Softmax parameter τ was increased to 0.1, the initial *Q*-values were lowered to 0.01, and the learning rate value α used for updating the *Q*-values was lowered to 0.05. In addition, as noted above, the IM agent selected an observer group on every odd-numbered episode, and then received a reward value after the subsequent even-numbered episode. Training of the PNs continued, as in Simulation 1, for all episodes.

### Results

Figure 6D presents the mean preference values for the five observer groups in Simulation 2, as a function of episode number. These values were analyzed following the same analytical strategy described in Simulation 1. A key finding from the analysis is that the range of preference values was considerably narrower than the pattern observed in Simulation 1. In addition, although we predicted that the COG samples from the infant observer group would have the highest initial preference values, this preference was not as robust as we anticipated. In particular, there was a marginally-significant preference for the infant observer group (*p* < 0.06) between episodes 1 and 50. Between episodes 50 and 100, there was no longer a significant difference between the infant and adult observers, though the two real observer groups had significantly higher preference values than the artificial observer groups (*p* < 0.01). This pattern maintained through episode 150. For the next 100 episodes (150–250) there was no significant difference between the five groups. Between episode 250 and 300, the leading preference shifted to the saliency observer group. This pattern persisted through the remaining episodes, although as Figure 6D illustrates, the preference values for the entropy observer group increased toward the end of training.

In contrast to Simulation 1, in which a clear preference for one of the observer groups was matched by a tendency for the corresponding group to also be selected consistently by the IM agent, there was a comparatively narrower preference pattern in Simulation 2, and as Figure 6E illustrates, also lack of a clear selection pattern. Indeed, the proportion of times each group was selected in Simulation 2 continued to fluctuate throughout the entire simulation.

Finally, Figure 6F presents the RMSE (pooled over observer groups) generated by the PNs over 500 episodes. In contrast to Figure 6C, the error rate declined more slowly in Simulation 2. There are several factors that may have contributed to this pattern. First, as noted above, the IM agent continued to explore until the end of Simulation 2, while in Simulation 1, exploratory selection of the sub-optimal observer groups ended on average by the 300th episode. Another contributing factor is that the relative differences in the five *Q*-values were smaller in Simulation 2, which also increased the chances of exploratory selections. Indeed, as we expected, there was no sustained “winner,” but rather, a series of shifts from one observer group to another.

However, it should be noted the second observer group that became preferred by the IM agent (i.e., after episode 250) was *not* the adult observer group, as we predicted. Instead, as Figure 6D illustrates, it was instead the saliency observer group. This result raises an important and interesting property of the reward function used in Simulation 2. In particular, note that the saliency observer group is the *least* preferred in Simulation 1, which is ostensibly due to having the largest initial prediction errors. Nevertheless, these initially high prediction errors may have helped to make the saliency observer group stand out in Simulation 2, as the COG samples from this group presumably provided the second-best opportunity for the IM agent to optimize its learning progress.

## General discussion

We provided an artificial agent with the opportunity to select among five sets of visual-exploration patterns, and then reinforced the agent for selecting COG samples that were either the easiest to learn (Simulation 1), or afforded the largest improvements in learning (Simulation 2), as estimated by a prediction-learning model. The agent was intrinsically-motivated, in the sense that it was not solving an explicit task—such as locating an object in a visual scene or comparing two images—but rather, it was rewarded for how well it learned (or more accurately, how well it selected a set of training images together with an artificial neural network that learned the set).

The pattern of findings from two simulation studies confirmed the first of three predictions, and partially confirmed the second. First, in Simulation 1—where the reward function was based on minimizing prediction errors—we found that the IM agent showed a consistent preference for learning from the COG image samples that were produced by human infants, rather than those produced by human adults, or those from three groups of artificial observers. Second, in Simulation 2 we predicted that infants' COG image samples would initially be preferred, and that the IM agent would then switch its preference to the adult observer group. While the first half of the prediction was confirmed, there were two qualifications: (a) the initial preference for the infant observer group was only marginally significant, and (b) this preference soon gave way to a collective preference for both the infant and adult COG image samples—that is, a preference for the real observer groups over the artificial observer groups. We also did not observe a clear switch to the adult observer group. Instead and contrary to our third prediction, the second preference “wave” in Simulation 2 was for the saliency observer group. While the data collected in the present study may not provide a comprehensive explanation for this result, we note below that our previous work highlights the important role of image salience, and may ultimately provide some insight into the pattern of findings in Simulation 2.

There are a number of implications for understanding development, as well as important questions, which are raised by these findings. First, our results suggest that if (1) prediction-learning and future-oriented actions play a central role in early visual development, and (2) infants are intrinsically-motivated to fine-tune and improve their ability to predict or forecast upcoming events, then the gaze patterns produced by 9-month-olds are well-suited to achieving both of those goals, compared to the gaze patterns of adults or the artificial observers that we generated. However, this finding also raises the question: what are the features of 9-month-olds' gaze patterns that make their COG samples easier to learn than those of other observers?

The kinematic analyses presented in Figure 3 suggest that how infants distribute their gaze over space may provide an important clue to answering this question. One possibility is that because 9-month-olds tend to have less-disperse gaze patterns than adults, and to shift their gaze a shorter distance, the resulting COG samples they produce tend to be more homogenous, and therefore, easier to learn. Alternatively, it may be the case that infants have the *a priori* goal of generating easily-learnable gaze patterns, and as a result, they therefore, tend to produce more compact scanpaths, with shorter gaze shifts between fixations. An essential step toward addressing this “chicken-and-egg” question is to collect gaze samples from a wider range of infants (e.g., 3- and 6-month-olds) and to evaluate the model when those additional COG samples are included. Another approach is to pit gaze-travel distance against local/global similarity, by using carefully-designed test images, in which there is high variability at the local level, with sets of highly-similar regions that are spaced relatively far apart.

A second issue suggested by our findings is what the developmental pattern will look like when the gaze data from younger infants are included. For example, should the agent prefer 3-month-olds' COG samples over those from 9-month-olds? In principle, with data from infants between birth and 12 months, our intuition is to expect an inverted U-shaped developmental pattern, in which gaze data from very young infants is poorly-controlled and therefore, highly unpredictable. We would then expect maximally-predictable COG samples between 3 and 4 months, and then an increasing trend afterwards of gradually less and less predictable gaze patterns. Fortunately, this is an empirical question that can be tested without any major modifications to our model.

Finally, a third question is whether the pattern of results—in particular, the shift that we observed during Simulation 2—can be interpreted as implying a *developmental pattern*. This is a difficult question to answer, as the timescale of the simulation reflects learning in an artificial agent, and does not map directly onto the infant-developmental timeline. Nevertheless, we might “read off” the results from Simulation 2 as suggesting that an initial strategy for visual exploration during infancy is to first focus on producing relatively dense clusters of fixations (i.e., like those produced by the two real-observer groups), which then shift toward becoming more widely distributed, and in particular, increasingly sensitive to the presence of salient regions in the visual scene. While this issue remains an open question, our prior work demonstrates that image saliency is an important factor that successfully accounts for infants' performance on a number of perceptual tasks (e.g., Schlesinger et al., 2007, 2011, 2012).

There are also a number of ways that our current approach can be improved. First, it is important to note that the PNs were trained offline—that is, the networks were trained to predict gaze sequences that had already been collected or generated. A disadvantage of this method is that any changes that occur in the agent cannot be propagated back to the observer groups. In other words, while the agent influences the amount of training time that each PN receives, it cannot influence how the COG samples are produced. An alternative and perhaps more-informative design would be for the choices of the agent to have an impact on the COG sampling process itself. Indeed, such a mechanism could be designed so that the production of eye movements in the artificial model is linked to the choices of the agent. However, there is no obvious way in which a similar connection could also be made between the agent and a live observer.

A second limitation of our model is that five different PNs were employed, which might be interpreted to suggest that infants' generate multiple sets of parallel predictors during visual exploration and then sample among them. While we remain agnostic to the specific cognitive structures or architectures exploited by human infants during visual exploration, a more elegant solution on the computational side would be to employ a single, unified predictor that learns over a range of sampling strategies (e.g., Schmidhuber, 2010).

Finally, a third issue concerns the models of the artificial observers, and in particular, the procedure used to transform the saliency and entropy maps into sequences of simulated eye movements. A key difference between the artificial and real observers is that the artificial observers tended to produce more disperse fixations, and return to previously-fixated locations less often than the human infants and adults. This issue can be addressed by imposing a theoretical energy or metabolic “cost” to the simulated eye movements, which is proportional to the size of the saccade. In addition, we can also continue to tune and improve the IOR mechanism, perhaps by modifying the decay rate, so that inhibition for previously-fixated locations decreases more rapidly. Another promising approach is to “yoke” the simulated gaze data to the actual moment-to-moment eye movements produced by real observers, so that kinematic measures such as fixation duration or saccade size are matched across the real and artificial data sets.

We conclude by noting that our work thus far takes advantage of machine-learning methods—in particular, the set of learning algorithms and architectures used to study intrinsic motivation in natural and artificial systems—as a means toward the goal of understanding visual development in human infants. Nevertheless, it is important to stress that the influence also runs in the other direction, that is, what lessons can be taken from our approach that might prove useful to the design of robots and artificial agents? One interesting insight is that our findings are consistent with the idea of “starting small” (e.g., Elman, 1993; Schlesinger et al., 2000): in other words, infants' gaze patterns may provide an advantageous starting point for learning in a naïve agent, relative to more-experienced observers such as adults. As we continue to extend and elaborate our model, in particular with data from younger infants, we anticipate that other important lessons for designing and developing artificial agents will continue to emerge.

### Conflict of interest statement

The authors declare that the research was conducted in the absence of any commercial or financial relationships that could be construed as a potential conflict of interest.
